# GC-IMS data on the discrimination between geographic origins of olive oils

**DOI:** 10.1016/j.dib.2022.108730

**Published:** 2022-11-08

**Authors:** Joscha Christmann, Sascha Rohn, Philipp Weller

**Affiliations:** a Institute for Instrumental Analytics and Bioanalysis, Mannheim University of Applied Sciences, Paul-Wittsack-Straße 10, 68163 Mannheim, Germany; b Hamburg School of Food Science, University of Hamburg, Grindelallee 117, 20146 Hamburg, Germany; c Department of Food Chemistry and Analysis, Institute of Food, Technology and Food Chemistry, Technische Universität Berlin, TIB 4/3-1, Gustav-Meyer-Allee 25, 13355 Berlin, Germany

**Keywords:** Non-target screening, Headspace, Food fraud detection, Chemometrics

## Abstract

Gas chromatography hyphenated with ion mobility spectrometry (GC-IMS) is an emerging benchtop technique for sensitive and selective detection of volatile organic compounds. It is commonly used for non-target screening (NTS) of complex sample materials, such as food products.

Resulting spectra are used as “fingerprints” for multivariate chemometric data analysis to extract information. This has been successfully applied in the field of food fraud detection in several published studies.

The presented dataset contains GC-IMS measurements of extra virgin olive oil samples from Spain, Italy, and Greece. It allows classification and class modelling to differentiate geographic origins and was used in the associated publication *gc-ims-tools, a new Python package for chemometric analysis of GC-IMS data* (https://doi.org/10.1016/j.foodchem.2022.133476) as an example to demonstrate the functionality.

## Specifications Table

**Table:** 

Subject	Analytical Chemistry
Specific subject area	Non-target screening, Food fraud detection, Chemometrics multivariate data analysis
Type of data	Binary files (*.mea format G.A.S. Sensorsysteme m.b.H., Dortmund, Germany)
How the data were acquired	Olive oil samples were analysed by headspace GC-IMS. The setup includes an Agilent 6890N gas chromatograph (Agilent Technologies, Palo Alto, USA), coupled with a standalone ion mobility spectrometer (Gesellschaft für Analytische Sensorsysteme mbH, Dortmund, Germany). Measurements were carried out in triplicates.
Data format	Raw
Description of data collection	53 olive oils from Spain (20), Italy (20) and Greece (13) of extra virgin quality, harvested during the 2014/2015 season, were kindly supplied by Coop Switzerland (Basel, Switzerland). Authenticity was verified by the supplier by chemical and isotope analysis.
Data source location	Mannheim University of Applied Sciences Institute for Instrumental Analysis and Bioanalytics Faculty of Biotechnology 68163 Mannheim, Germany
Data accessibility	Weller, Philipp; Christmann, Joscha (2022), “Olive oil geography by GC-IMS analysis”, Mendeley Data, V3, doi: 10.17632/fr9t5fkkvz.3 URL: https://data.mendeley.com/datasets/fr9t5fkkvz
Related research article	J. Christmann, S. Rohn, P. Weller, gc-ims-tools – A new Python package for chemometric analysis of GC–IMS data, Food Chem. 224 (2022) 133476. https://doi.org/10.1016/j.foodchem.2022.133476.

## Value of the Data

• The data are of benefit for analytical chemists working on food fraud detection and/or chemometric data analysis.
• The dataset has been analysed extensively in previous publications and can therefore be used to benchmark new statistical methods or implementations against known results [1,2].
• The data can be used as additional training data for machine learning models to predict the geographical origin of extra virgin olive oils (data fusion or with other GC-IMS data).
• The dataset is used for explaining the chemometric analysis of GC-IMS data in a tutorial of the *gc-ims-tools* Python package [3].

## 1 Data Description

The dataset consists of 53 authentic extra virgin olive oil samples from Greece (13), Italy (20) and Spain (20). Measurements were made in duplicates or triplicates, therefore a total of 151 GC-IMS spectra are included. Each measurement is stored in the binary .mea file by G.A.S Dortmund mbH. The data is raw i.e., without any preprocessing applied, and in its original file format because it was used as the tutorial dataset for the free and open-source Python package *gc-ims-tools* which provides those functionalities. *gc-ims-tools* was presented in detail in the related research article [3]. Alternatively the vendors commercial software VOCal or the free, vendor independent software OpenChrom (version 1.5) can be used to read and visualize GC-IMS data [4]. The benefit of using the binary format over CSV data is the significantly lower file size.

**Fig. 1:**
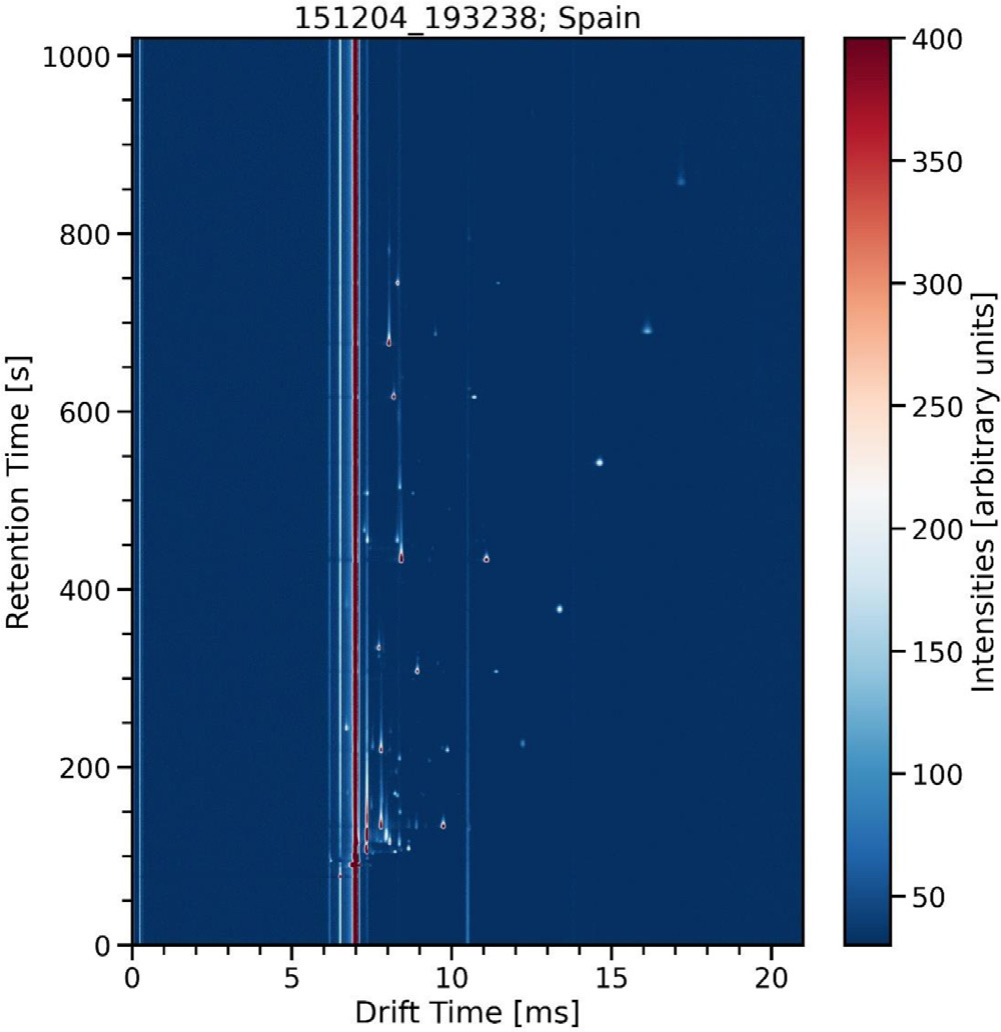
Heat map visualization of an olive oil GC-IMS spectrum.

Each measurement contains a matrix of intensity values of size (6939, 3150). The first dimension features the GC retention time ranging from 0 to 1020 s, the second is the IMS drift time ranging from 0 to 21 ms. A heat map visualization of one of the measurements of the Spanish olive oil sample O-015 one of the spectra is exemplarily shown in Fig. 1.

## 2 Experimental Design, Materials, and Methods

Olive oils of extra virgin quality, harvested during the 2014/2015 season, were kindly supplied by Coop Switzerland (Basel, Switzerland). Analytical standards were purchased at the highest available quality (≥98 %). 2-Acetylpyridine (Sigma-Aldrich Chemie GmbH, Taufkirchen, Germany) was used as internal standard. Ultrapure water was purified in-house, using a Milli-Q water-purification system (Millipore, Bedford, MA, USA). Anhydrous sodium chloride was obtained from VWR International GmbH (Darmstadt, Germany). The GC-IMS setup includes an Agilent 6890N gas chromatograph (Agilent Technologies, Palo Alto, USA), coupled with a standalone ion mobility spectrometer (Gesellschaft für Analytische Sensorsysteme mbH, Dortmund, Germany). A CombiPal GC autosampler (CTC Analytics AG, Zwingen Switzerland) with a headspace sampling unit and a 2.5 mL syringe (Gerstel GmbH, Mülheim, Germany) was used for sample injection. The separation was carried out on a NB-225 capillary column 25 m x 0.32 mm x 0.25 µm from HNU-Nordion Ltd. (Oy, Finland) with a constant nitrogen (99.99 % purity) flow of 1.5 ml/min. The split/splitless injector was held at 150 °C with a split ratio of 1:30 and a headspace liner with 1.2 mm i.d. was used (Agilent, Waldbronn, Germany). The initial oven temperature was 40 °C for 2 min before it was ramped with 8 °C/min to 150 °C and held constant for an additional 10 min. The IMS unit was mounted with a heated transfer line (150 °C) and operated with a drift gas flow of 150 mL/min at 90 °C. For the analysis 1 g of olive oil was spiked with 18 µL of 2-acetylpyridine stock solution (1008 mg/L) and mixed with 1 mL of saturated sodium chloride solution. After an incubation period of 15 min at 45 °C 500 µL headspace volume was injected [1].

## Ethics Statements

Not applicable.

## CRediT authorship contribution statement

**Joscha Christmann:** Data curation, Writing – original draft, Visualization, Software. **Sascha Rohn:** Writing – review & editing. **Philipp Weller:** Writing – review & editing, Funding acquisition, Supervision, Conceptualization.

## Declaration of Competing Interest

The authors declare no competing interests.

## Data Availability

Olive oil geography by GC-IMS analysis (Reference data) (Mendeley Data).
